# *BRCA1/2 *Reversion Mutations in Japanese Patients with Metastatic Breast Cancer Progressing on Olaparib: OLIVE (WJOG15321B)

**DOI:** 10.1007/s12282-026-01855-2

**Published:** 2026-04-10

**Authors:** Hitomi Sakai, Rika Kusumoto-Matsuo, Mari Hosonaga, Kazuki Nozawa, Manabu Futamura, Yuko Tanabe, Toru Mukohara, Kazuhiro Shiraishi, Tatsuya Toyama, Hiroyuki Yasojima, Noriyuki Watanabe, Hideki Sakai, Tsutomu Iwasa, Nobumichi Takeuchi, Hiroshi Tada, Kazuko Sakai, Natsuko Chiba, Toshimi Takano, Junji Tsurutani

**Affiliations:** 1 Advanced Cancer Translational Research Institute, Showa Medical University, 1-5-8 Hatanodai, Shinagawa-ku, Tokyo 142-8555 Japan; 2 Breast Oncology Center, Cancer Institute Hospital of the Japanese Foundation for Cancer Research, Tokyo, Japan; 3 Department of Breast Oncology, Aichi Cancer Center, Nagoya, Japan; 4 Department of Breast Surgery, Gifu University Hospital, Gifu, Japan; 5 Department of Medical Oncology, Toranomon Hospital, Tokyo, Japan; 6 Department of Oncology, National Cancer Center Hospital East, Kashiwa, Japan; 7 Department of Medical Oncology, NHO Nagoya Medical Center, Nagoya, Japan; 8 Department of Breast Surgery, Nagoya City University, Nagoya, Japan; 9 Department of Surgery, Breast Oncology, NHO Osaka National Hospital, Osaka, Japan; 10 Department of Breast and Endocrine Surgery, Osaka International Cancer Institute, Osaka, Japan; 11 Department of Medical Oncology, Hyogo Cancer Center, Akashi, Japan; 12 Department of Medical Oncology, Kindai University Faculty of Medicine, Osaka, Japan; 13 Department of Oncology, Ina Central Hospital, Ina, Japan; 14 Department of Surgery, Division of Breast and Endocrine Surgery, Tohoku University Hospital, Sendai, Japan; 15 Department of Genome Biology, Kindai University Faculty of Medicine, Osaka, Japan; 16 Department of Cancer Biology, Institute of Development, Aging and Cancer (IDAC), Tohoku University, Sendai, Japan

**Keywords:** olaparib, PARP inhibitor, reversion mutation, *BRCA1/2*, resistance

## Abstract

**Background:**

A key resistance mechanism to PARP inhibitors is the development of *BRCA1/2* reversion mutations that restore the protein function.

**Methods:**

Patients with metastatic breast cancer and germline *BRCA1/2* mutations were recruited from 14 institutions and treated with olaparib. Targeted next-generation sequencing was performed on circulating tumor DNA (ctDNA) extracted from blood collected at base line and progression on olaparib (Guardant360). The primary objective was to evaluate the frequency of *BRCA1/2* reversion mutations in this group.

**Results:**

From November 2021 to October 2023, 60 patients were enrolled. The median age was 50.5 years (32–85). Of these, 12 (20.0%) had germline *BRCA1* mutation and 48 (80.0%) had germline *BRCA2* mutation. The median number of prior chemotherapies was 2 (0–7). Two patients had received platinum. Among patients with tumor progression on olaparib who had available ctDNA results, *BRCA1/2* reversion mutations were identified in 25.0% (2/8; 95% CI 3.2–65.1%) with germline *BRCA1* mutation and 23.5% (8/34, 95% CI 10.7–41.2%) with germline *BRCA2* mutation. In one patient, *BRCA2* reversion mutations were found in ctDNA seven months before radiological confirmation of progression on olaparib and again after progression. Among the ten cases with *BRCA1/2* reversion mutations, all but one case involved secondary indels on primary indel sites. Reversion mutations were not observed in the C-terminal regions of *BRCA1/2*.

**Conclusion:**

*BRCA1/2* reversion mutations were detected in ctDNA as a mechanism of resistance to olaparib. Certain genomic regions and mutation types appeared to be particularly prone to reversion mutations.

**Clinical trial registration:**

UMIN000046007.

**Supplementary Information:**

The online version contains supplementary material available at 10.1007/s12282-026-01855-2.

## Introduction

Poly (adenosine diphosphate–ribose) polymerase (PARP) inhibitors have revolutionized the treatment of patients with breast cancer harboring germline *BRCA1/2* mutation [1, 2]. However, resistance to PARP inhibitors can occur. Mechanisms of resistance to PARP inhibitors are classified into three categories: BRCA-dependent homologous recombination repair (HR) activity restoration (e.g. *BRCA1/2* reversion mutation), BRCA-independent HR activity restoration (e.g. reversion mutations of *RAD51C*, *RAD51D* and *PALB2* and loss of 53BP1) [3, 4], and HR-independent mechanisms (e.g. SLFN11 inactivation and overexpression of ABCC1 and ABCG2) [5, 6]. Secondary mutations that restore HR activity of BRCA1/2, known as reversion mutations, have been documented as a cause of resistance to PARP inhibitors and platinum [7, 8].

However, there are no studies reporting somatic *BRCA1/2* reversion mutations in circulating tumor DNA (ctDNA) in Japanese populations with metastatic breast cancer and germline *BRCA1/2* mutation. Several reports have suggested population-specific prevalence of *BRCA1/2* variants [9, 10]; therefore, it is meaningful to evaluate reversion mutations in the Japanese population. The objective of this study is to evaluate the frequency of *BRCA1/2* reversion mutations using ctDNA in Japanese patients with metastatic breast cancer harboring germline *BRCA1/2* mutation who received treatment with olaparib. Additionally, we intended to characterize the clinical and molecular features of cases with *BRCA1/2* reversion mutation.

### Patients and methods

Patients with metastatic breast cancer carrying germline *BRCA1/2* mutation were enrolled from 14 institutions and treated with olaparib. The study included patients who had not yet initiated olaparib (cohort 1), those who were receiving olaparib without evidence of disease progression (cohort 2), and those who had already completed olaparib therapy (cohort 3). Blood collection was performed at the baseline and end of treatment with olaparib. Treatment duration was defined as the time from the initiation of olaparib to the date of last dose. Study protocol was approved by the institutional review board (Approval number: 21–029-B) and was done in accordance with the Declaration of Helsinki and Ethical Guidelines for Medical and Biological Research Involving Human Subjects. Patients provided written informed consent before participation.

### Circulating tumor DNA analysis

Blood was collected using Streck Cell-Free tubes, while frozen samples of plasma previously collected in EDTA and obtained with patient consent were also permitted for the ctDNA analysis for four samples. Tumor specimens before and/or after olaparib were collected from a limited number of participants; however, the results are not reported here. Deidentified blood samples were sent to Guardant Health (California, US) and plasma were extracted for targeted next-generation sequencing of 74 cancer-related genes (Supplementary Table 1), mostly driver genes, using the Guardant360 [11, 12]. Putative germline mutations were determined by a betabionomial algorithm [13]. A *BRCA1/2* reversion mutation was defined as a base substitution changing a deleterious mutation to a non-deleterious mutation, an insertion or deletion (indel) restoring the open reading frame (ORF) disrupted by the deleterious mutation, or an in-frame deletion spanning and removing the deleterious mutation entirely.

### Statistical analysis

The median duration of treatment was estimated using the Kaplan–Meier method, and the 95% confidence interval (CI) was calculated accordingly. Statistical analysis was carried out using JMP Student Edition 19.

## Results

### Patients and blood samples

The enrollment period was from November 2021 to October 2023, during which 60 patients were registered. Among them, 20.0% of patients had germline *BRCA1* mutation and 80.0% of patients had germline *BRCA2* mutation. Two and 42 patients had prior platinum and anthracycline treatment, respectively (Supplementary Table 2).

Forty-five patients (75%) discontinued olaparib treatment during the observational period, all due to disease progression (Supplementary Fig. 1). Median treatment duration was 11.7 months (95% CI 9.0–13.1). Baseline blood samples were collected from all the patients (N = 60), and post-treatment blood samples were obtained from 42 patients. Tumor tissue analysis data are not included in this report.

### Incidence of *BRCA1/2* reversion mutations

No *BRCA1/2* reversion mutations were detected in any of the 13 pre-olaparib samples. Among those who discontinued olaparib due to tumor progression, reversion mutations occurred in 25.0% (2/8; 95% CI 3.2–65.1%) with germline *BRCA1* mutations and 23.5% (8/34, 95% CI 10.7–41.2%) with germline *BRCA*2 mutations. In total, the incidence was 23.8% (10/42; 95% CI 12.1–39.5%). Multiple reversion mutation clones in *BRCA2* were detected in a patient receiving olaparib (NG001), about 7 months before radiological progression. In this case, VAF of c.1403_1436del dramatically increased from 0.25% to 16.55%, much higher than that of the other reversion mutations, suggesting that this revertant clone may represent the dominant resistant population (Supplementary Fig. 5).

Median treatment duration was numerically longer in patients without detectable *BRCA1/2* reversion mutation in the post progression sample (N = 32) compared to those with detectable *BRCA1/2* reversion mutation (N = 10) (10.1 months [95%CI 8.1–11.8] versus 7.3 months [95% CI 3.6–8.3]) (Supplementary Fig. 2).

### The distribution of primary *BRCA1/2* mutation that underwent reversion

**Fig. 1:**
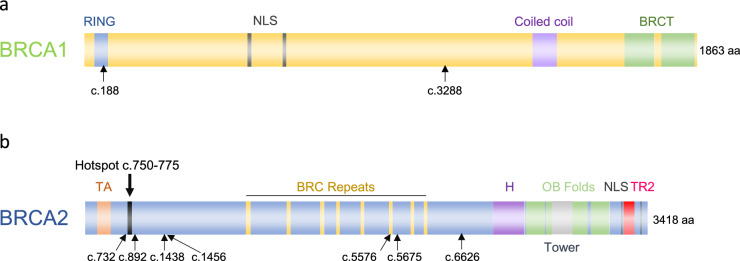
Distribution of primary mutation reverted in *BRCA1 and BRCA2*. **a** The positions of primary mutations are shown on the domain structure of *BRCA1* (n = 2). Coding DNA sequence (CDS) numbers are indicated. RING, Zinc finger domain; NLS, nuclear localization signal; BRCT, *BRCA1* C-terminal domain. **b** Same as in panel a but for *BRCA2* (n = 8). According to the report by Pettitt SJ et al., *BRCA2* c.750–775 was a frequent site of primary mutations in *BRCA2* reversion mutations, which is shown as “Hotspot” [19]. Primary mutation of 2 cases is located on the same position (c.6626). Abbreviations: TA, transcriptional activation domain; H, helical domain; OB Folds, oligonucleotide binding folds; Tower, tower domain; TR2, Rad51 binding domain

In cases with *BRCA1* reversion mutations, the primary mutations originated in two regions: one case in the Really Interesting New Gene (RING) domain, and the other between the nuclear localization signal (NLS) and the coiled-coil domain (Fig. 1a). For *BRCA2*, four cases had primary mutations located between the N-terminus and the BRC repeat, two cases within the BRC repeats, and two cases between the BRC repeats and the DNA-binding domain (DBD) (Fig. 1b).

### Type of mutations that cause reversion mutations

Among the ten cases with *BRCA1/2* reversion mutations, all but one cases involved secondary indels on primary indel sites (Table 1, Supplementary Fig. 3, Supplementary Fig. 4) and the one case harbored a secondary single nucleotide variant (SNV) on a primary SNV site, suggesting that the additional indels on the original indels were the major mechanisms of acquired resistance against olaparib through restoration of the homologous recombinant activity (Table 1).

**Table 1:** The frequency of different pathogenic mutation types

A. *BRCA1*				
	Primary mutations			
	Deletion	Insertion	SNV	Total
Allele numbers	1	0	1	2
%	50.0 (95%CI: 1.3–98.7)	0.0 (95%CI: 0.0–84.2)	50.0 (95%CI: 1.3–98.7)	100
	Secondary mutations			
Allele numbers	1	0	2	3
%	33.3 (95%CI: 0.8–90.6)	0.0 (95%CI: 0.0–70.8)	66.7 (95%CI: 9.4–99.2)	100

B. *BRCA2*				
	Primary mutations			
	Deletion	Insertion	SNV	Total
Allele numbers	5	3	0	8
%	62.5 (95%CI: 24.5–91.5)	37.5 (95%CI: 8.5–75.5)	0 (95%CI: 0–36.9)	100
	Secondary mutations			
Allele numbers	16	3	0	19
%	84.2 (95%CI: 60.4–96.6)	15.8 (95%CI: 3.4–39.6)	0.0 (95%CI: 0.0–17.6)	100

*CI* confidence interval

### Mutational landscape in post progression on PARP inhibitor

**Fig. 2:**
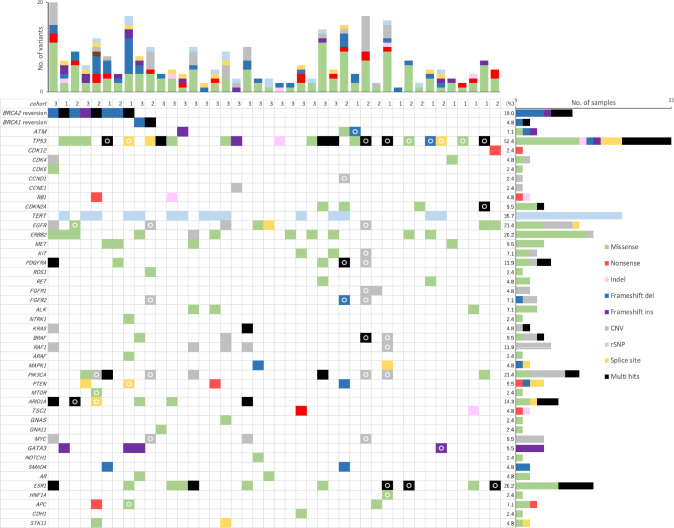
Analysis of ctDNA post progression on PARP inhibitors. Mutational landscape of ctDNA from 42 patients. Post-specific mutations are indicated by circles. Note that post-specific mutations cannot be determined in cohort 3. “Multi-hit” includes either the same or different types of classifications

Most post progression samples (41/42, 97.6%) harbored mutation(s) in genes other than *BRCA1/2*. Gene alterations of *TP53* (22/42, 52.4%), *TERT* (15/42, 35.7%), *ERBB2* (11/42, 26.2%), *ESR1* (11/42, 26.2%), *PIK3CA* (9/42, 21.4%) and *EGFR* (9/42, 21.4%) were most commonly observed in post progression ctDNA samples (Fig. 2).

Among patients whose paired samples were evaluable, frequency of post specific mutations were *TP53* (9/22, 40.9%), *TERT* (0/22, 0%), *ERBB2* (0/22,0%), *ESR1* (3/22,13.6%), *PIK3CA* (4/22, 18.2%), and *EGFR* (3/22, 13.6%). The relevance of these gene alterations is not clear if they contributed to resistance specific to the PARP inhibitor although they have been involved in drug resistance to certain therapies [14–16] and may have implications for combination therapy with olaparib (e.g., oral Selective Estrogen Receptor Degrader (SERD) or phosphoinositide 3-kinase (PI3K) inhibitors) [17, 18].

## Discussion

This study examined the frequency of *BRCA1/2* reversion mutations occurred in Japanese patients with metastatic breast cancer that had progressed on olaparib. Most participants had not previously received platinum-based treatments, which made it possible to evaluate how frequently reversion mutations appeared upon PARP inhibitor resistance in actual clinical samples of breast cancer.

*BRCA1/2* reversion mutations generally arise under selective pressure of homologous recombination deficiency (HRD)-targeted therapy, especially PARP inhibitors or platinum [19]. In this study, blood samples were collected from 40 patients either prior to treatme*nt* or during treatment—before clinical progression. Among them, the majority of participants (37; 92.5%) had a history of chemotherapy, with 27 (67.5%) receiving prior anthracycline and 2 (5.0%) receiving prior platinum-based therapy. Notably, no *BRCA1/2* reversion mutations were detected in the pretreatment blood samples from those patients. However, substantial patients have developed *BRCA1/2* reversion mutations after olaparib exposure, which indicates selective pressure of olaparib at least in part had played a role in inducing *BRCA1/2* reversion mutations. The emergence of multiple *BRCA1/2* reversion mutations—up to 8 mutations in the present study—indicates strong selective pressure by olaparib on tumor subclones [20]. In one case, *BRCA2* reversion mutation emerged during treatment and before clinical progression, and VAF of which increased after continuous olaparib exposure, suggesting strong selective pressure of olaparib induced expansion of resistance clone (Supplementary Fig. 5).

A recent study reported that *BRCA1*/*2* reversion mutations in ctDNA from 60% of patients with metastatic breast cancer at the time of disease progression on HRD-targeted therapy [21]. In contrast, studies including multiple cancer types have consistently shown lower frequencies, generally in the range of approximately 20–40% [22–24]. In our cohort, the frequency of the reversion mutations at disease progression on olaparib was approximately 24%. Several factors may have been attributed to discrepancy between OLIVE and a study by Harvey-Jones E, et al. [21]. First, patients’ backgrounds were different. For example, the prevalence of pathogenic germline *BRCA1* mutation was higher in the study by Harvey-Jones E, et al.; 46.8% [21]. Second, the incidence of prior platinum exposure was higher in the study by Harvey-Jones E, et al.; 74.5% received prior (neo)adjuvant chemotherapy and 21.3% prior platinum containing palliative chemotherapy [21]. Third, they utilized GuardantINFINITY as ctDNA assay, which has higher sensitivity than Guardant 360, enabling detection of low-frequency variants [21].

ctDNA analysis has several inherent limitations. First, the limit of detection for mutant allele fraction increased with lower amount of cell free DNA input. Second, large reversion mutations may not be detected. Third, determining cis/trans configuration can be challenging because ctDNA fragments are short (< 200 bp). Therefore, comparisons across studies require caution. On the contrary, ctDNA is a powerful tool in evaluating the evolution of resistant subclones. Further investigation in a larger cohort is necessary.

A previous study showed that the resultant *BRCA1* missense variant L63E in the RING domain by reversion mutation has HR activity [25]. Considering the data, the reversion mutation in *BRCA1* would have acquired HR activity and became resistant to PARP inhibitor in one case (JF014). The other *BRCA1* and *BRCA2* alterations by reversion mutation, except for SU006, were located in exon 10 of *BRCA1* and exon 10–11 of *BRCA2*. These regions are considered “coldspots”, that tolerate variation, and pathogenic missense variants are rarely observed [26]. Although functional relevance should be interpreted cautiously, all revertant have the homologous recombination activity and resulted in resistance to PARP inhibitor.

Previous studies identified regions more likely to acquire reversion mutations, as well as regions less prone to reversion events [19, 24]. Consistent with our study, *BRCA2* reversion mutations were frequently observed in BRC repeats or the N-terminal region, between the PALB2-interacting domain and the BRC repeats. In contrast, reversion mutations were relatively rare in the C-terminal region, encompassing the DNA-binding domains (DBD), possibly because this region is highly conserved and critical for homologous recombinant function [27, 28]. Particularly, RAD51-binding TR2 motif in the C-terminal region is essential in protection and restart of stalled replication forks [29]. In *BRCA1*, the BRCA1 C-terminal (BRCT) domain and RING domain were found to be the hotspots [24]. In our cohort, only two cases with germline *BRCA1* mutations were identified to be a revertant, which located them outside of the hotspots and limited the comparison with previous reports across these domains due to the small sample size. A subgroup analysis of the phase III PAOLA-1/ENGOT-ov25 trial indicated that maintenance olaparib and bevacizumab was especially effective for advanced high-grade ovarian cancer patients with DBD mutations in *BRCA1*/*2* [30]. These findings suggested that *BRCA1* and *BRCA2* pathogenic variants in the DBDs were less prone to acquiring reversion mutations, which may have allowed tumor cells to stay homologous recombination deficient leading to durable responses to PARP inhibitors.

Consistent with previous reports, *BRCA1/2* frameshift indels were more prone to reversion than SNV [31]. Previous studies have indicated that restoration of the reading frame can be achieved by diverse secondary indels generated through error-prone classical nonhomologous end joining (NHEJ) or microhomology-mediated end-joining (MMEJ), allowing partial recovery of homologous recombination function without requiring exact sequence restoration [31].

This study is subject to several major limitations. Firstly, the sample size is relatively small. The caution should be used for the interpretation of the contents. A future integrated analysis across multiple cohorts would enhance the robustness of the current findings. Secondly, we didn’t evaluate the clinical endpoints (e.g. progression free survival, objective response rate, overall survival) with olaparib. Therefore, we couldn’t correlate *BRCA1/2* reversion mutations with those endpoints. A previous study showed that pre-treatment *BRCA1/2* reversion mutation inversely correlated with response to rucaparib in patients with ovarian cancer [32]. As increasing number of patients with metastatic breast cancer currently receive platinum-based therapy prior to olaparib, evaluating the correlation of pre-treatment existence of *BRCA1/2* reversion mutation and response might be meaningful. Thirdly, we cannot exclude co-existence of other resistant mechanisms to olaparib. Guardant 360, which we utilized in the study, didn’t cover HR related genes including *PALB2*, *RAD51C*, or *RAD51D* and genes implicated in resistance to PARP inhibitors such as *TP53BP1*, *RIF1* and *PAXIP1.* Fourth, we didn’t collect detailed treatment histories other than prior platinum and anthracycline exposure. Although two patients had received platinum, we don’t have information on whether it was administered in the perioperative or metastatic setting. Finally, all patients received olaparib. PARP inhibitors vary in their ability to trap; olaparib shows relatively moderate trapping activity [33]. It is unclear whether PARP trapping level among the various PARP inhibitors impacts on the emerging mutations in patients with that treatment. Further studies are warranted. Despite the limitations, our findings are of clinical value for further development of precision medicine in patients with *BRCA1/2* through monitoring of reversion mutations with ctDNA and devising strategies to overcome resistance.

## Conclusions

*BRCA1/2* reversion mutations were detected in ctDNA as a mechanism of resistance to olaparib. Certain genomic regions and mutation types appeared to be prone to reversion mutations. In addition, the presence of alternative resistance mechanisms besides *BRCA1/2* reversion was yet to be elucidated.

## Supplementary Information

Below is the link to the electronic supplementary material.

Supplementary file1 (XLSX 13 KB)

Supplementary file2 (DOCX 25 KB)

Supplementary file3 (PPTX 42 KB)

Supplementary file4 (PPTX 68 KB)

Supplementary file5 (PPTX 46 KB)

Supplementary file6 (PPTX 93 KB)

Supplementary file7 (PPTX 196 KB)

## Data Availability

The data that support the findings of this study are available from the corresponding author upon reasonable request.
