# Supplementary material for: BRCA1/2 Reversion Mutations in Japanese Patients with Metastatic Breast Cancer Progressing on Olaparib: OLIVE (WJOG15321B)
Source: Breast Cancer. 2026 Apr 10;33(3):790–7. doi: 10.1007/s12282-026-01855-2 (PMC13124753; doi:10.1007/s12282-026-01855-2)
Supplement: Supplementary file 2 — Supplementary file2 (DOCX 25 KB) [file 12282_2026_1855_MOESM2_ESM.docx]

**Supplementary Table 1. Patients’ characteristics**

|  | *BRCA1* mutation  N = 12 | *BRCA2* mutation  N = 48 | All  N = 60 |
| --- | --- | --- | --- |
| **Age, median (range)** | 40.5 (32-85) | 51.0 (38-84) | 50.5 (32-85) |
| **Stage, N (%)** |  |  |  |
| Recurrent | 11 (91.7) | 40 (66.7) | 51 (85.0) |
| De novo stage Ⅳ | 1 (8.3) | 8 (13.3) | 9 (15.0) |
| **Histology, N (%)** |  |  |  |
| IDC | 11 (91.7) | 41 (85.4) | 52 (86.7) |
| ILC | 0 (0) | 1 (2.1) | 1 (1.7) |
| Other | 1 (8.3) | 6 (12.5) | 7 (11.7) |
| **Number of prior chemotherapy regimens,**  **median (range)** | 3 (0-4) | 2 (0-7) | 2 (0-7) |
| **Prior platinum, N (%)** | 1 (8.3) | 1 (2.1) | 2 (3.3) |
| **Prior anthracycline, N (%)** | 9 (75.0) | 33 (68.8) | 42 (70.0) |

Abbreviations; N, number; IDC, invasive ductal carcinoma; ILD, invasive lobular carcinoma
