# Supplementary material for: BRCA1/2 Reversion Mutations in Japanese Patients with Metastatic Breast Cancer Progressing on Olaparib: OLIVE (WJOG15321B)
Source: Breast Cancer. 2026 Apr 10;33(3):790–7. doi: 10.1007/s12282-026-01855-2 (PMC13124753; doi:10.1007/s12282-026-01855-2)
Supplement: Supplementary file 3 — Supplementary file3 (PPTX 42 KB) [file 12282_2026_1855_MOESM3_ESM.pptx]

## Slide 1
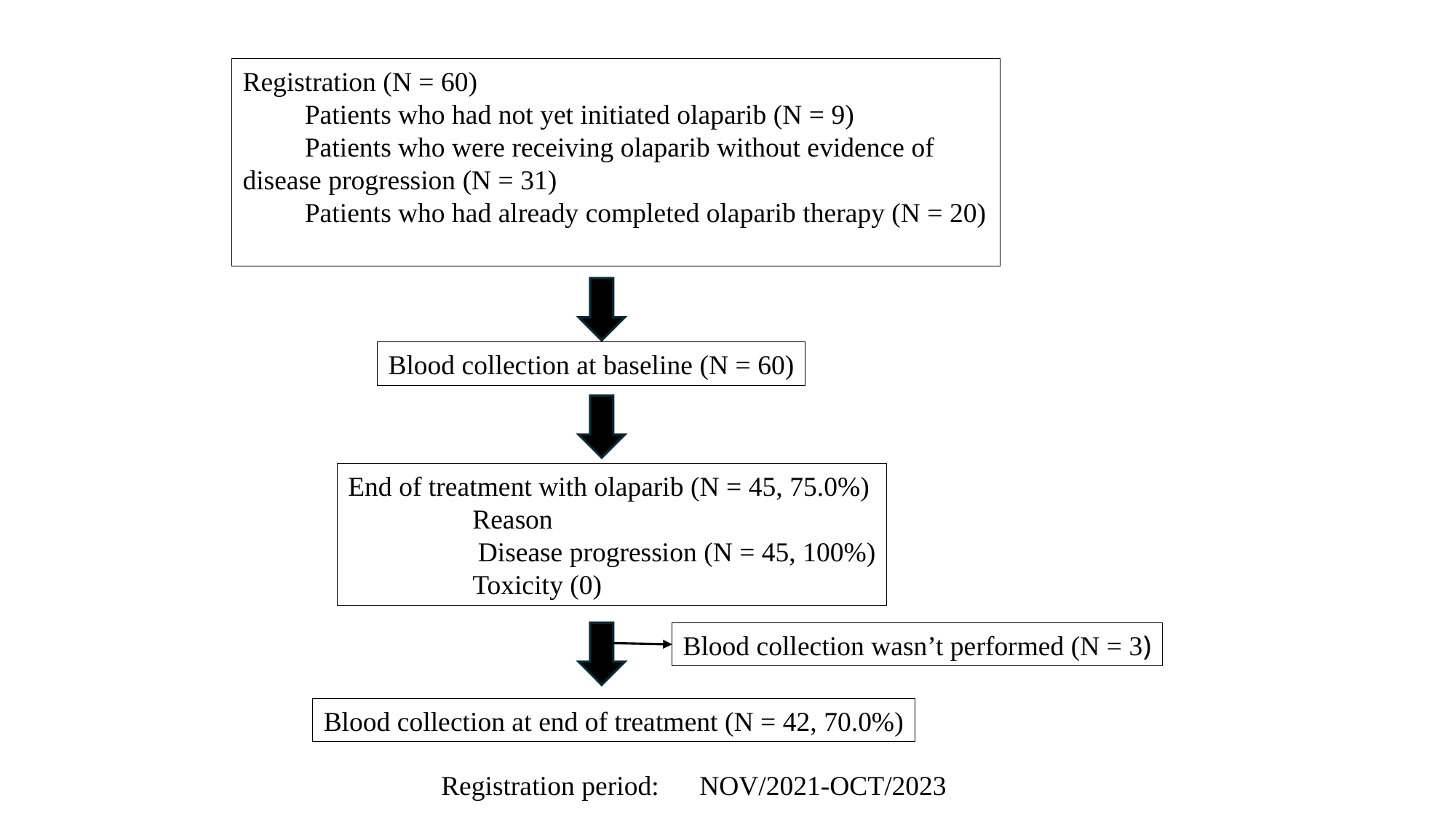

Registration (N = 60)
 Patients who had not yet initiated olaparib (N = 9)
 Patients who were receiving olaparib without evidence of disease progression (N = 31)
 Patients who had already completed olaparib therapy (N = 20)
Blood collection at baseline (N = 60)
End of treatment with olaparib (N = 45, 75.0%)
 Reason
　　　　 Disease progression (N = 45, 100%)
 Toxicity (0)
Blood collection wasn’t performed (N = 3)
Blood collection at end of treatment (N = 42, 70.0%)
Registration period:　NOV/2021-OCT/2023
