# Supplementary figures and images for: BRCA1/2 Reversion Mutations in Japanese Patients with Metastatic Breast Cancer Progressing on Olaparib: OLIVE (WJOG15321B)
Source: Breast Cancer. 2026 Apr 10;33(3):790–7. doi: 10.1007/s12282-026-01855-2 (PMC13124753; doi:10.1007/s12282-026-01855-2)

## Slide 1
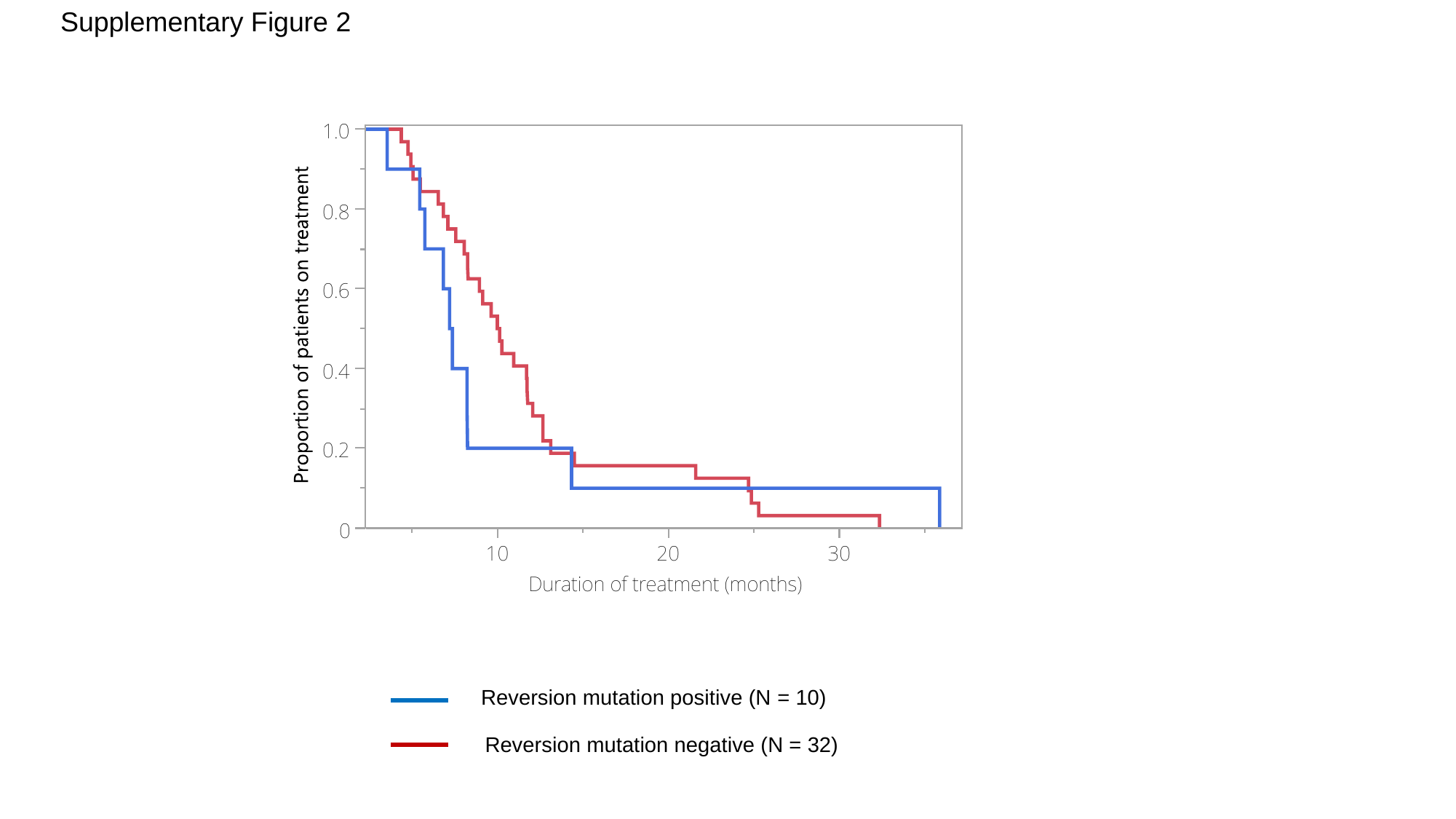

# Supplementary Figure 2
Reversion mutation positive (N = 10)
Reversion mutation negative (N = 32)

Supplement: Supplementary file 4 — Supplementary file4 (PPTX 68 KB) [file 12282_2026_1855_MOESM4_ESM.pptx]
