# Supplementary material for: BRCA1/2 Reversion Mutations in Japanese Patients with Metastatic Breast Cancer Progressing on Olaparib: OLIVE (WJOG15321B)
Source: Breast Cancer. 2026 Apr 10;33(3):790–7. doi: 10.1007/s12282-026-01855-2 (PMC13124753; doi:10.1007/s12282-026-01855-2)
Supplement: Supplementary file 5 — Supplementary file5 (PPTX 46 KB) [file 12282_2026_1855_MOESM5_ESM.pptx]

## Slide 1
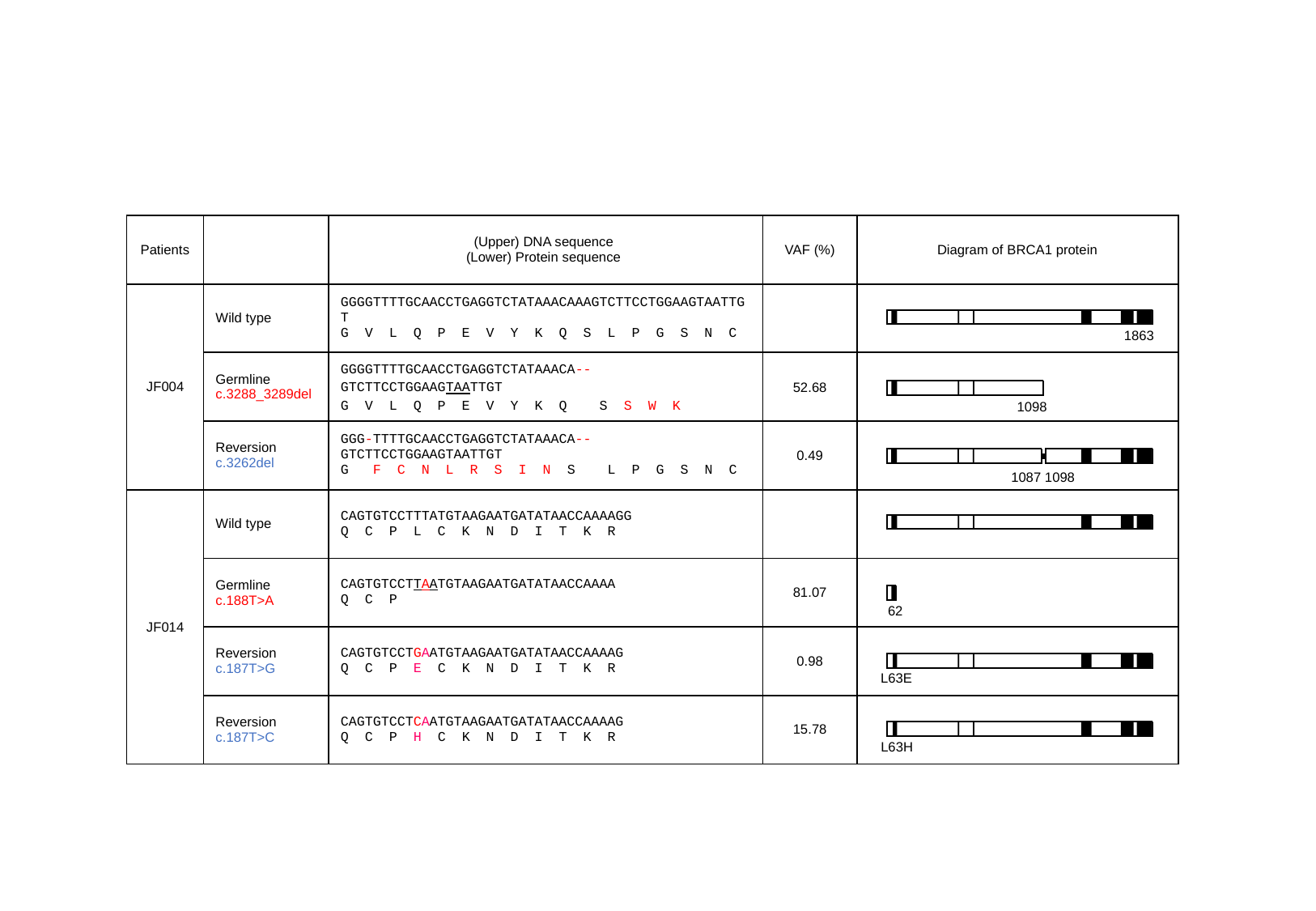

| Patients | | (Upper) DNA sequence (Lower) Protein sequence | VAF (%) | Diagram of BRCA1 protein |
| --- | --- | --- | --- | --- |
| JF004 | Wild type | GGGGTTTTGCAACCTGAGGTCTATAAACAAAGTCTTCCTGGAAGTAATTGT G V L Q P E V Y K Q S L P G S N C | | |
| | Germline c.3288\_3289del | GGGGTTTTGCAACCTGAGGTCTATAAACA--GTCTTCCTGGAAGTAATTGT G V L Q P E V Y K Q S S W K | 52.68 | |
| | Reversion c.3262del | GGG-TTTTGCAACCTGAGGTCTATAAACA--GTCTTCCTGGAAGTAATTGT G F C N L R S I N S L P G S N C | 0.49 | |
| JF014 | Wild type | CAGTGTCCTTTATGTAAGAATGATATAACCAAAAGG Q C P L C K N D I T K R | | |
| | Germline c.188T>A | CAGTGTCCTTAATGTAAGAATGATATAACCAAAA Q C P | 81.07 | |
| | Reversion c.187T>G | CAGTGTCCTGAATGTAAGAATGATATAACCAAAAG Q C P E C K N D I T K R | 0.98 | |
| | Reversion c.187T>C | CAGTGTCCTCAATGTAAGAATGATATAACCAAAAG Q C P H C K N D I T K R | 15.78 | |
1863
1098
1087 1098
62
L63E
L63H
