# Supplementary material for: BRCA1/2 Reversion Mutations in Japanese Patients with Metastatic Breast Cancer Progressing on Olaparib: OLIVE (WJOG15321B)
Source: Breast Cancer. 2026 Apr 10;33(3):790–7. doi: 10.1007/s12282-026-01855-2 (PMC13124753; doi:10.1007/s12282-026-01855-2)
Supplement: Supplementary file 6 — Supplementary file6 (PPTX 93 KB) [file 12282_2026_1855_MOESM6_ESM.pptx]

## Slide 1
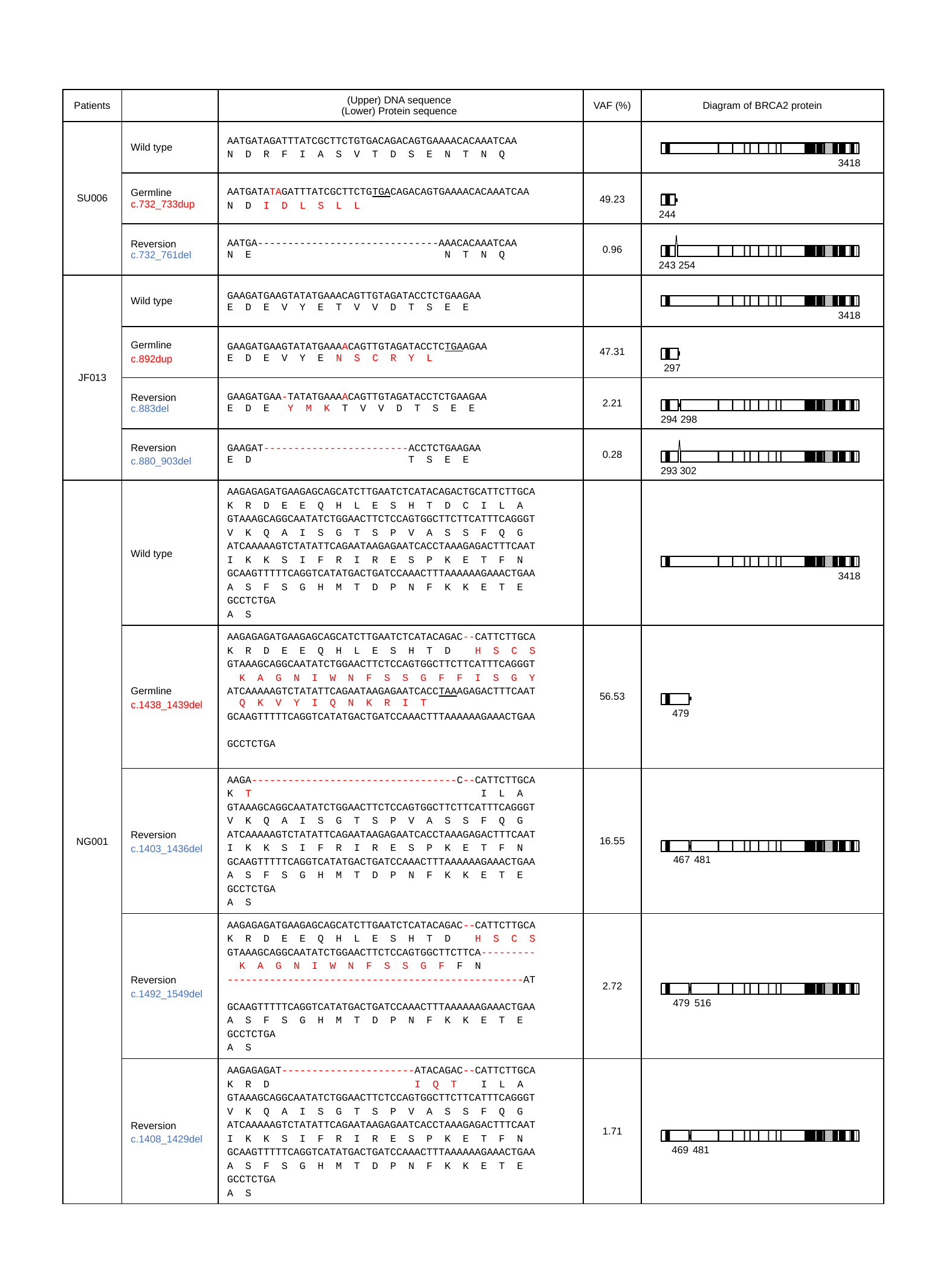

| Patients | | (Upper) DNA sequence (Lower) Protein sequence | VAF (%) | Diagram of BRCA2 protein |
| --- | --- | --- | --- | --- |
| SU006 | Wild type | AATGATAGATTTATCGCTTCTGTGACAGACAGTGAAAACACAAATCAA N D R F I A S V T D S E N T N Q | | |
| | Germline c.732\_733dup | AATGATATAGATTTATCGCTTCTGTGACAGACAGTGAAAACACAAATCAA N D I D L S L L | 49.23 | |
| | Reversion c.732\_761del | AATGA------------------------------AAACACAAATCAA N E N T N Q | 0.96 | |
| JF013 | Wild type | GAAGATGAAGTATATGAAACAGTTGTAGATACCTCTGAAGAA E D E V Y E T V V D T S E E | | |
| | Germline c.892dup | GAAGATGAAGTATATGAAAACAGTTGTAGATACCTCTGAAGAA E D E V Y E N S C R Y L | 47.31 | |
| | Reversion c.883del | GAAGATGAA-TATATGAAAACAGTTGTAGATACCTCTGAAGAA E D E Y M K T V V D T S E E | 2.21 | |
| | Reversion c.880\_903del | GAAGAT------------------------ACCTCTGAAGAA E D T S E E | 0.28 | |
| NG001 | Wild type | AAGAGAGATGAAGAGCAGCATCTTGAATCTCATACAGACTGCATTCTTGCA K R D E E Q H L E S H T D C I L A GTAAAGCAGGCAATATCTGGAACTTCTCCAGTGGCTTCTTCATTTCAGGGT V K Q A I S G T S P V A S S F Q G ATCAAAAAGTCTATATTCAGAATAAGAGAATCACCTAAAGAGACTTTCAAT I K K S I F R I R E S P K E T F N GCAAGTTTTTCAGGTCATATGACTGATCCAAACTTTAAAAAAGAAACTGAA A S F S G H M T D P N F K K E T E GCCTCTGA A S | | |
| | Germline c.1438\_1439del | AAGAGAGATGAAGAGCAGCATCTTGAATCTCATACAGAC--CATTCTTGCA K R D E E Q H L E S H T D H S C S GTAAAGCAGGCAATATCTGGAACTTCTCCAGTGGCTTCTTCATTTCAGGGT K A G N I W N F S S G F F I S G Y ATCAAAAAGTCTATATTCAGAATAAGAGAATCACCTAAAGAGACTTTCAAT Q K V Y I Q N K R I T GCAAGTTTTTCAGGTCATATGACTGATCCAAACTTTAAAAAAGAAACTGAA GCCTCTGA | 56.53 | |
| | Reversion c.1403\_1436del | AAGA----------------------------------C--CATTCTTGCA K T I L A GTAAAGCAGGCAATATCTGGAACTTCTCCAGTGGCTTCTTCATTTCAGGGT V K Q A I S G T S P V A S S F Q G ATCAAAAAGTCTATATTCAGAATAAGAGAATCACCTAAAGAGACTTTCAAT I K K S I F R I R E S P K E T F N GCAAGTTTTTCAGGTCATATGACTGATCCAAACTTTAAAAAAGAAACTGAA A S F S G H M T D P N F K K E T E GCCTCTGA A S | 16.55 | |
| | Reversion c.1492\_1549del | AAGAGAGATGAAGAGCAGCATCTTGAATCTCATACAGAC--CATTCTTGCA K R D E E Q H L E S H T D H S C S GTAAAGCAGGCAATATCTGGAACTTCTCCAGTGGCTTCTTCA--------- K A G N I W N F S S G F F N -------------------------------------------------AT GCAAGTTTTTCAGGTCATATGACTGATCCAAACTTTAAAAAAGAAACTGAA A S F S G H M T D P N F K K E T E GCCTCTGA A S | 2.72 | |
| | Reversion c.1408\_1429del | AAGAGAGAT----------------------ATACAGAC--CATTCTTGCA K R D I Q T I L A GTAAAGCAGGCAATATCTGGAACTTCTCCAGTGGCTTCTTCATTTCAGGGT V K Q A I S G T S P V A S S F Q G ATCAAAAAGTCTATATTCAGAATAAGAGAATCACCTAAAGAGACTTTCAAT I K K S I F R I R E S P K E T F N GCAAGTTTTTCAGGTCATATGACTGATCCAAACTTTAAAAAAGAAACTGAA A S F S G H M T D P N F K K E T E GCCTCTGA A S | 1.71 | |
3418
244
243
254
3418
297
294
298
293
302
3418
479
467
481
479
516
469
481

## Slide 2
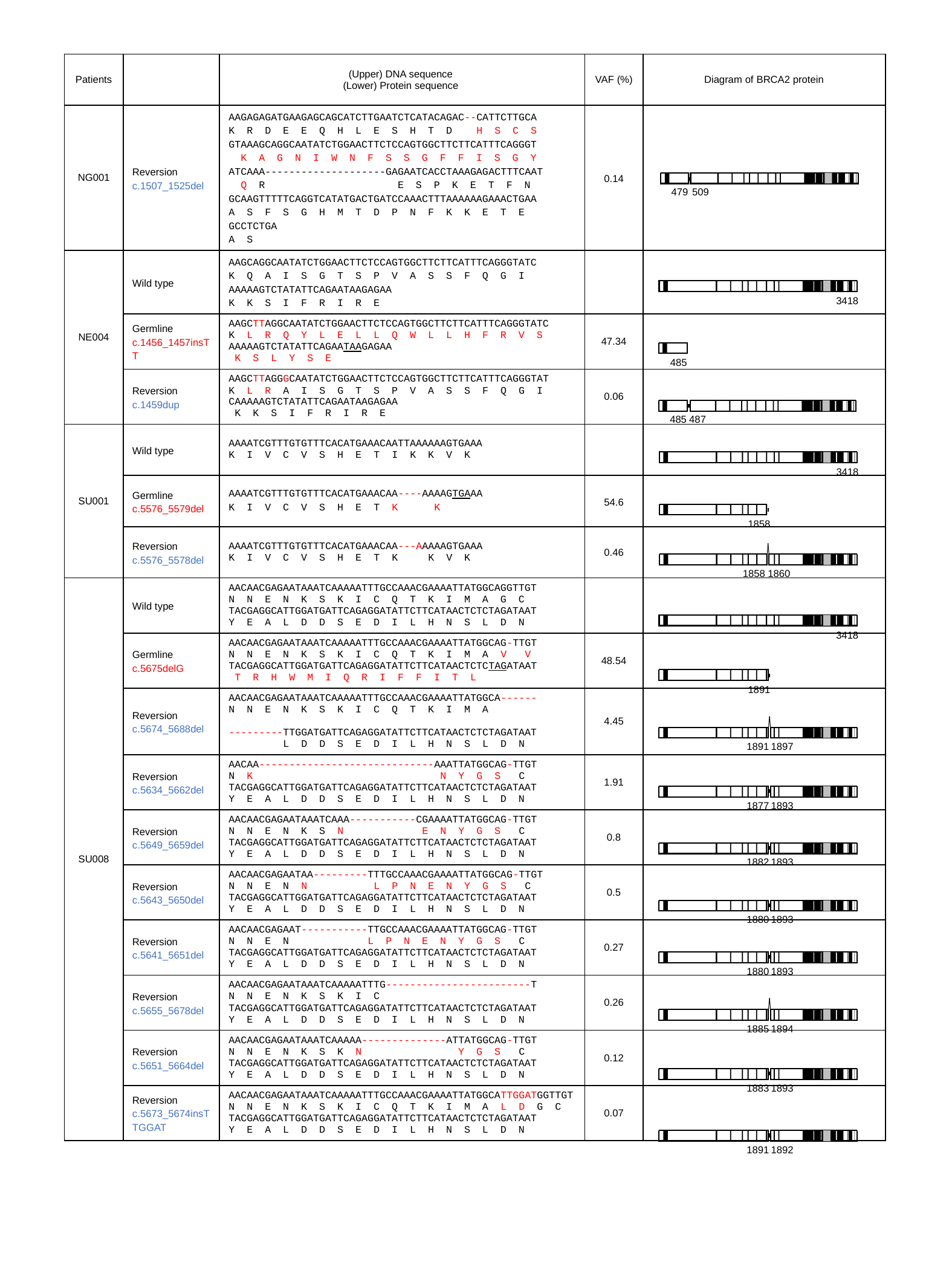

| Patients | | (Upper) DNA sequence (Lower) Protein sequence | VAF (%) | Diagram of BRCA2 protein |
| --- | --- | --- | --- | --- |
| NG001 | Reversion c.1507\_1525del | AAGAGAGATGAAGAGCAGCATCTTGAATCTCATACAGAC--CATTCTTGCA K R D E E Q H L E S H T D H S C S GTAAAGCAGGCAATATCTGGAACTTCTCCAGTGGCTTCTTCATTTCAGGGT K A G N I W N F S S G F F I S G Y ATCAAA--------------------GAGAATCACCTAAAGAGACTTTCAAT Q R E S P K E T F N GCAAGTTTTTCAGGTCATATGACTGATCCAAACTTTAAAAAAGAAACTGAA A S F S G H M T D P N F K K E T E GCCTCTGA A S | 0.14 | |
| NE004 | Wild type | AAGCAGGCAATATCTGGAACTTCTCCAGTGGCTTCTTCATTTCAGGGTATC K Q A I S G T S P V A S S F Q G I AAAAAGTCTATATTCAGAATAAGAGAA K K S I F R I R E | | |
| | Germline c.1456\_1457insTT | AAGCTTAGGCAATATCTGGAACTTCTCCAGTGGCTTCTTCATTTCAGGGTATC K L R Q Y L E L L Q W L L H F R V S AAAAAGTCTATATTCAGAATAAGAGAA K S L Y S E | 47.34 | |
| | Reversion c.1459dup | AAGCTTAGGGCAATATCTGGAACTTCTCCAGTGGCTTCTTCATTTCAGGGTAT K L R A I S G T S P V A S S F Q G I CAAAAAGTCTATATTCAGAATAAGAGAA K K S I F R I R E | 0.06 | |
| SU001 | Wild type | AAAATCGTTTGTGTTTCACATGAAACAATTAAAAAAGTGAAA K I V C V S H E T I K K V K | | |
| | Germline c.5576\_5579del | AAAATCGTTTGTGTTTCACATGAAACAA----AAAAGTGAAA K I V C V S H E T K K | 54.6 | |
| | Reversion c.5576\_5578del | AAAATCGTTTGTGTTTCACATGAAACAA---AAAAAGTGAAA K I V C V S H E T K K V K | 0.46 | |
| SU008 | Wild type | AACAACGAGAATAAATCAAAAATTTGCCAAACGAAAATTATGGCAGGTTGT N N E N K S K I C Q T K I M A G C TACGAGGCATTGGATGATTCAGAGGATATTCTTCATAACTCTCTAGATAAT Y E A L D D S E D I L H N S L D N | | |
| | Germline c.5675delG | AACAACGAGAATAAATCAAAAATTTGCCAAACGAAAATTATGGCAG-TTGT N N E N K S K I C Q T K I M A V V TACGAGGCATTGGATGATTCAGAGGATATTCTTCATAACTCTCTAGATAAT T R H W M I Q R I F F I T L | 48.54 | |
| | Reversion c.5674\_5688del | AACAACGAGAATAAATCAAAAATTTGCCAAACGAAAATTATGGCA------ N N E N K S K I C Q T K I M A ---------TTGGATGATTCAGAGGATATTCTTCATAACTCTCTAGATAAT L D D S E D I L H N S L D N | 4.45 | |
| | Reversion c.5634\_5662del | AACAA-----------------------------AAATTATGGCAG-TTGT N K N Y G S C TACGAGGCATTGGATGATTCAGAGGATATTCTTCATAACTCTCTAGATAAT Y E A L D D S E D I L H N S L D N | 1.91 | |
| | Reversion c.5649\_5659del | AACAACGAGAATAAATCAAA-----------CGAAAATTATGGCAG-TTGT N N E N K S N E N Y G S C TACGAGGCATTGGATGATTCAGAGGATATTCTTCATAACTCTCTAGATAAT Y E A L D D S E D I L H N S L D N | 0.8 | |
| | Reversion c.5643\_5650del | AACAACGAGAATAA---------TTTGCCAAACGAAAATTATGGCAG-TTGT N N E N N L P N E N Y G S C TACGAGGCATTGGATGATTCAGAGGATATTCTTCATAACTCTCTAGATAAT Y E A L D D S E D I L H N S L D N | 0.5 | |
| | Reversion c.5641\_5651del | AACAACGAGAAT-----------TTGCCAAACGAAAATTATGGCAG-TTGT N N E N L P N E N Y G S C TACGAGGCATTGGATGATTCAGAGGATATTCTTCATAACTCTCTAGATAAT Y E A L D D S E D I L H N S L D N | 0.27 | |
| | Reversion c.5655\_5678del | AACAACGAGAATAAATCAAAAATTTG------------------------T N N E N K S K I C TACGAGGCATTGGATGATTCAGAGGATATTCTTCATAACTCTCTAGATAAT Y E A L D D S E D I L H N S L D N | 0.26 | |
| | Reversion c.5651\_5664del | AACAACGAGAATAAATCAAAAA--------------ATTATGGCAG-TTGT N N E N K S K N Y G S C TACGAGGCATTGGATGATTCAGAGGATATTCTTCATAACTCTCTAGATAAT Y E A L D D S E D I L H N S L D N | 0.12 | |
| | Reversion c.5673\_5674insTTGGAT | AACAACGAGAATAAATCAAAAATTTGCCAAACGAAAATTATGGCATTGGATGGTTGT N N E N K S K I C Q T K I M A L D G C TACGAGGCATTGGATGATTCAGAGGATATTCTTCATAACTCTCTAGATAAT Y E A L D D S E D I L H N S L D N | 0.07 | |
479
509
3418
485
485
487
3418
1858
1858
1860
3418
1891
1891
1897
1877
1893
1882
1893
1880
1893
1880
1893
1885
1894
1883
1893
1891
1892

## Slide 3
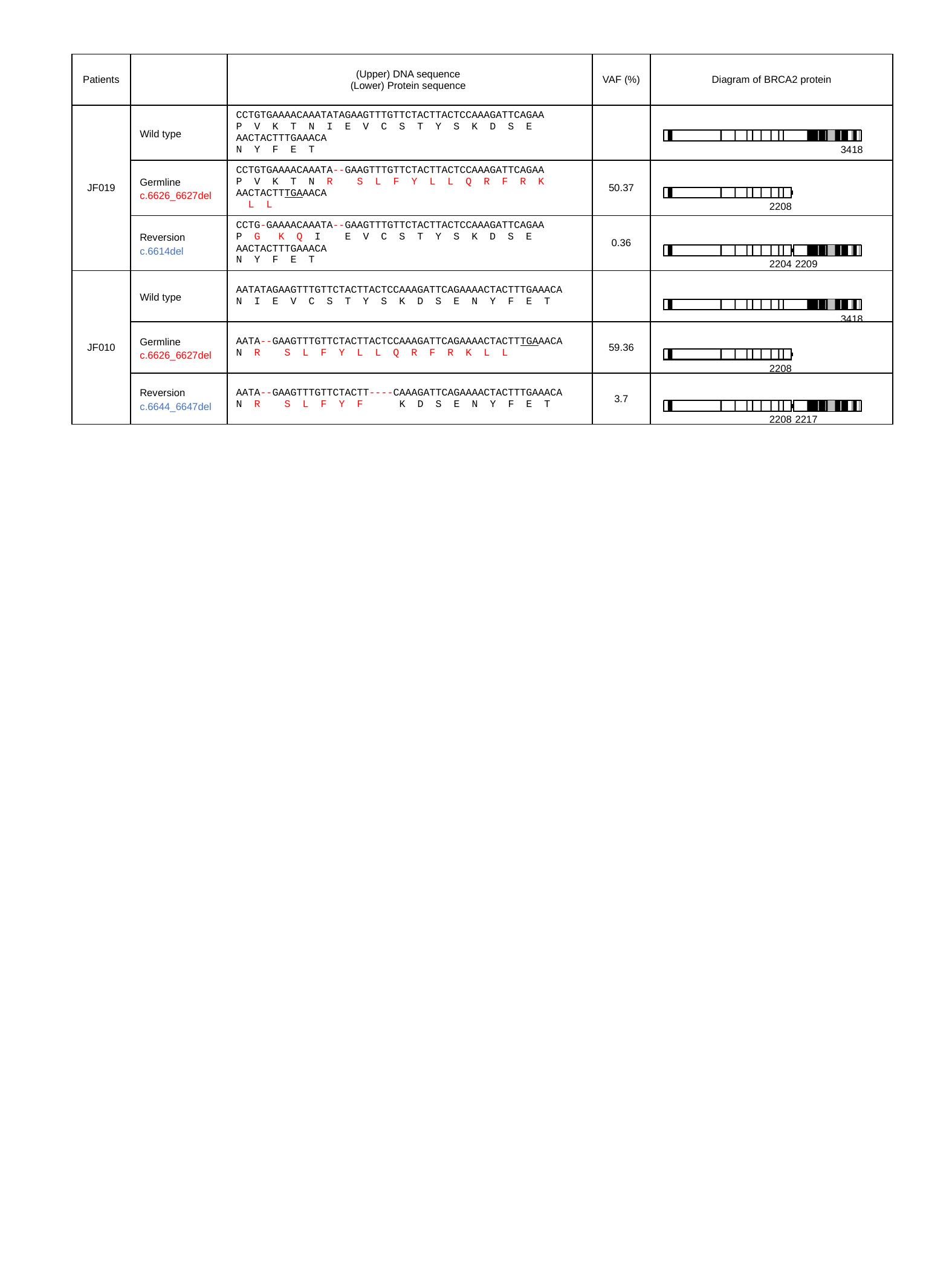

| Patients | | (Upper) DNA sequence (Lower) Protein sequence | VAF (%) | Diagram of BRCA2 protein |
| --- | --- | --- | --- | --- |
| JF019 | Wild type | CCTGTGAAAACAAATATAGAAGTTTGTTCTACTTACTCCAAAGATTCAGAA P V K T N I E V C S T Y S K D S E AACTACTTTGAAACA N Y F E T | | |
| | Germline c.6626\_6627del | CCTGTGAAAACAAATA--GAAGTTTGTTCTACTTACTCCAAAGATTCAGAA P V K T N R S L F Y L L Q R F R K AACTACTTTGAAACA L L | 50.37 | |
| | Reversion c.6614del | CCTG-GAAAACAAATA--GAAGTTTGTTCTACTTACTCCAAAGATTCAGAA P G K Q I E V C S T Y S K D S E AACTACTTTGAAACA N Y F E T | 0.36 | |
| JF010 | Wild type | AATATAGAAGTTTGTTCTACTTACTCCAAAGATTCAGAAAACTACTTTGAAACA N I E V C S T Y S K D S E N Y F E T | | |
| | Germline c.6626\_6627del | AATA--GAAGTTTGTTCTACTTACTCCAAAGATTCAGAAAACTACTTTGAAACA N R S L F Y L L Q R F R K L L | 59.36 | |
| | Reversion c.6644\_6647del | AATA--GAAGTTTGTTCTACTT----CAAAGATTCAGAAAACTACTTTGAAACA N R S L F Y F K D S E N Y F E T | 3.7 | |
3418
2208
2204
2209
3418
2208
2208
2217
