# Supplementary material for: BRCA1/2 Reversion Mutations in Japanese Patients with Metastatic Breast Cancer Progressing on Olaparib: OLIVE (WJOG15321B)
Source: Breast Cancer. 2026 Apr 10;33(3):790–7. doi: 10.1007/s12282-026-01855-2 (PMC13124753; doi:10.1007/s12282-026-01855-2)
Supplement: Supplementary file 7 — Supplementary file7 (PPTX 196 KB) [file 12282_2026_1855_MOESM7_ESM.pptx]

## Slide 1
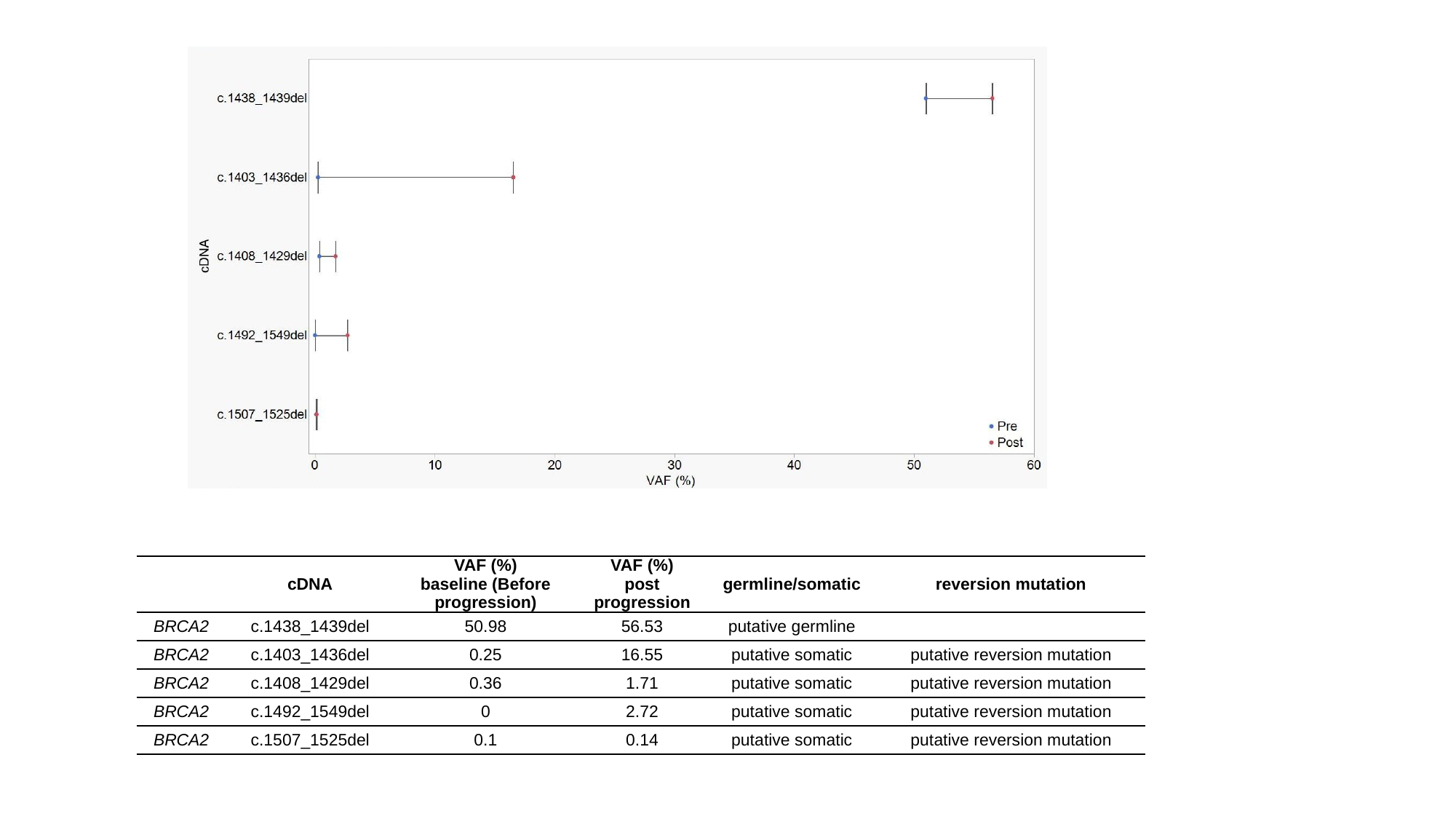

| | cDNA | VAF (%) baseline (Before progression) | VAF (%) post progression | germline/somatic | reversion mutation |
| --- | --- | --- | --- | --- | --- |
| BRCA2 | c.1438\_1439del | 50.98 | 56.53 | putative germline | |
| BRCA2 | c.1403\_1436del | 0.25 | 16.55 | putative somatic | putative reversion mutation |
| BRCA2 | c.1408\_1429del | 0.36 | 1.71 | putative somatic | putative reversion mutation |
| BRCA2 | c.1492\_1549del | 0 | 2.72 | putative somatic | putative reversion mutation |
| BRCA2 | c.1507\_1525del | 0.1 | 0.14 | putative somatic | putative reversion mutation |
